# What Can We Do to Improve Relations between Clinicians and Managers?

**Published:** 1991-03

**Authors:** Peter Sims

**Affiliations:** Director of Public Health, N. Devon Health Authority

## Abstract

This paper reports on another way in which the outstanding Management problems of the 1990's have been addressed in one particular District in the South West. It indicates the experience of using a particular model over two years and indicates that this could be a useful way forward, both in the District and the Trust of Northern Devon, but also in other Districts facing similar difficulties.


### What Can We Do To Improve Relationships Between Clinicians and Managers?

**Peter Sims, MSc, MFCM, MRCGP**
Director of Public Health, N. Devon Health Authority.

### SUMMARY

This paper reports on another way in which the outstanding Management problems of the 1990's have been addressed in one particular District in the South West. It indicates the experience of using a particular model over two years and indicates that this could be a useful way forward, both in the District and the Trust of Northern Devon, but also in other Districts facing similar difficulties.

### INTRODUCTION

The White Paper, the new GP contract, and organisational change within Social Services have been opposed by most doctors. The inbuilt confrontation which is inevitable in a situation where Doctors use resources and Managers try to control them, has been emphasized by this new situation. Thus, many Managers see their promotions and career linked to the White Paper, while the majority of Clinicians see little wrong with the present Health Service apart from lack of funding. The British Medical Association, their professional organisation, urges them not to co-operate. The result only too easily is confrontation, argument and stalemate.

The situation in North Devon mirrors that at national level with the problem perhaps magnified by the relatively small numbers of Consultants and Managers looking after a scattered population. Arguably, North Devon in the past has provided an excellent Health Service; on the hospital site, Consultant led and Consultant run (there are few junior staff above SHO level in the District); General Practice is seen as an attractive option with strong competition for few vacancies; while for Managers North Devon has acquired an excellent record with opportunity for experience, advancement and promotion to senior posts in other parts of the Country.

The Director of Public Health Medicine arrived one month after the White Paper was published, and over the next few months became aware of the confrontation between Doctors and Managers when a bid for an independent Trust for the whole of North Devon was put forward. His previous experience, working as an academic and with groups in an educational model, suggested that bringing together Consultants, General Practitioners and Managers in a group situation where the dynamic of the group could be used appropriately was worthwhile in an attempt to break the deadlock. In a group situation, Doctors and Managers could meet on neutral ground rather than in the usual confrontation of "You've closed by beds". "I had to because you were overspending and budgets were out of control". It was important that the emphasis of the seminars was educational and exploratory thus, the Chairman of the Medical Executive Committee, who had been anxious about both the political and emotional temperature of the District; was involved as well as the Post Graduate Tutors in General Practice and Hospital Medicine. They were happy to support this venture and advised on structure and timetable. Thus, 13 Consultants, 13 General Practitioners and 13 Managers were selected informally and invited personally, with a letter signed by the two Clinical Tutors and the Director of Public Health Medicine to attend a series of five Seminars through the winter months. The condition of acceptance was that people agreed to attend all five seminars. The topics of the five seminars were decided in advance as:

1. Management
2. Information
3. Finance
4. Consumerism/Audit
5. The Future of the NHS

Of the people invited, most agreed to come, despite the difficulty in timing for General Practitioners. When the final timetable was sent out each person was also given a resource pack which would be built upon through the five seminars. There would be a working paper to be read in advance appropriate to a particular session. Afterwards a synoptic paper indicating the main lines of the seminar was then circulated. Thus, over the five sessions an information pack would be collected giving some of the key ideas within the subject and their implication for North Devon.

### THE FORMAT

The evening was arranged so that following tea the meeting started at 5.30 p.m. with an introductory talk lasting no more than 15 minutes from an invited speaker. This introduction would be essentially a scene setting exercise, building on the main points in the handout for that day. After this outline introduction, the large group would break into three smaller groups of some 10–15 people who would examine an agreed problem in a group situation for one hour. The membership of each group remained constant throughout the five seminars and each group was a mixture of Managers and Clinicians. It was hoped that in this way a group dynamic would be developed. On the whole the groups worked, although sometimes problems of leadership and personality surfaced which detracted from the progress that a group could make. The three groups then reconvened at 6.45 p.m. for debriefing and the session closed promptly at 7.00 p.m.

Each seminar was headed by a guest from outside North Devon with particular skills and knowledge in the relevant topic. Thus,

| | |
|---|---|
| 1. Management: | Mr. Stephen Cang |
| 2. Information: | Dr. Derek Pheby |
| 3. Finance: | Mr. Ed McNally |
| 4. Consumerism/Audit: | Dr. Charles Shaw |
| 5. The Future of the NHS: | Mr. Stephen Cang/<br>Dr. Alastair Mason |

### RESULTS

Overall 16 General Practitioners, 15 Hospital Doctors and 15 Managers attended one or more sessions. The mean attendance of the group as a whole was 3.65 out of a maximum of 5 with no real difference between the numbers attending from the three major participants. There was little or no drop off in attendance by participants and all sessions were attended by at least 30 individuals.

In trying to assess each seminar participants were asked to indicate their feelings, before and after the meetings so that their relative interest, enthusiasm, tiredness, etc could be indicated and how that changed in the course of the seminar. They were also asked to indicate the quality of the session and the knowledge gained, the effectiveness of the group work and the overall score for the day.

Following the seminars, the participants were asked to give again their overall evaluation of the series. Thus, the second survey which has been scored as one for yes, minus one for no and nought for don't know's gives an overall indication of the seminars, what people felt they would gain from it, the structure

**Table 1: Attendance at Seminars**

| | | | | | | TOTAL of 1 or more attendances | TOTAL attendances | MEAN attendance |
|---|---|---|---|---|---|---|---|---|
| Seminar | 1 | 2 | 3 | 4 | 5 | | | |
| Managers | 11 | 8 | 11 | 11 | 12 | 15 | 53 | 3.53 |
| GPs | 11 | 11 | 12 | 11 | 12 | 16 | 57 | 3.56 |
| Hospital Doctors | 13 | 11 | 13 | 11 | 10 | 15 | 58 | 3.87 |
| TOTAL | 35 | 30 | 36 | 33 | 34 | 46 | 168 | 3.68 |

**Table 2: Total Numbers of Attendances per Individual**

| | x1 | x2 | x3 | x4 | x5 | TOTAL |
|---|---|---|---|---|---|---|
| Managers | – | 1 | 7 | 5 | 2 | 53 |
| GPs | 1 | 3 | 2 | 6 | 4 | 57 |
| Hospital Doctors | – | 2 | 2 | 7 | 4 | 58 |
| TOTAL | 1 | 6 | 11 | 18 | 10 | 168 |

for the future, the use or otherwise of handouts. Paradoxically minus scores here indicate that the participants did not find the seminars too demanding or handouts too time consuming.

### DISCUSSION

The consensus view coming from the evaluations was that this project had been successful and overall had achieved the key objectives which were particularly:

1. To increase mutual awareness and understanding;
2. To enable exchange of views to occur;
3. To learn new things;
4. To provide a resource pack;
5. To develop new thinking for the future.

The fact that attendance stayed up and that the scores of evaluation were reasonably high would support this view. Interestingly, the task of the last session of the course was "The Future" and here each person was asked to write in 500 words his or her own view about their own future and that of the NHS in the next five years. These contributions were then collated to identify common threads and problems. This collation was used as a basis for their work on the last evening. This exercise showed both the coming together between the Doctors and the Managers and also some of the things that separated them.

Once the sessions were over and all the evaluations were completed, each participant received a report on this work and the result of the evaluations.There was strong support for this in North Devon. Key lessons learned, which may be applicable elsewhere are:

1. To run the groups under an educational umbrella with the backup of the Clinical Tutors in General Practice and Hospital Medicine;
2. To start punctually and end on time;

**Mean Score**

| Seminar | No Responding | Quality | Knowledge Gained | Group Work | Overall | Total |
|---|---|---|---|---|---|---|
| 1 **Management** | | | | | | |
| Managers | 9 | 3.6 | 2.6 | 3.4 | 3.5 | |
| GPs | 9 | 3.6 | 2.8 | 2.7 | 3.1 | 3.1 |
| Hospital Doctors | 10 | 3.2 | 2.3 | 3.1 | 2.9 | |
| 2 **Information** | | | | | | |
| Managers | 3 | 2.7 | 2.0 | 2.0 | 2.0 | |
| GPs | 8 | 3.0 | 2.6 | 2.9 | 3.0 | 2.7 |
| Hospital Doctors | 6 | 3.5 | 2.2 | 3.1 | 3.0 | |
| 3 **Finance** | | | | | | |
| Managers | 7 | 3.9 | 3.0 | 3.6 | 3.9 | |
| GPs | 6 | 3.5 | 2.7 | 2.7 | 3.1 | 3.2 |
| Hospital Doctors | 9 | 3.4 | 2.1 | 2.9 | 3.0 | |
| 4 **Consumerism/Audit** | | | | | | |
| Managers | 4 | 3.3 | 3.0 | 3.0 | 3.5 | |
| GPs | 5 | 3.8 | 3.0 | 3.2 | 3.6 | 3.3 |
| Hospital Doctors | 4 | 4.0 | 3.2 | 2.8 | 3.0 | |
| 5 **Future Changes** | | | | | | |
| Managers | 7 | 3.9 | 3.3 | 4.1 | 4.3 | |
| GPs | 4 | 4.0 | 3.0 | 3.8 | 4.0 | 3.3 |
| Hospital Doctors | 2 | 4.0 | 2.5 | 3.3 | 3.5 | |
| **Overall Scores** | | | | | | |
| Managers | | 3.5 | 2.8 | 3.2 | 3.2 | |
| GPs | | 3.6 | 2.9 | 3.1 | 3.4 | |
| Hospital Doctors | | 3.6 | 2.4 | 3.0 | 3.1 | |

| | Managers | GPs & Community Physicians | Hospital Doctors | TOTAL |
|---|---|---|---|---|
| TOTAL REPLIES | 12 | 9 | 8 | +29 |
| Did you find the Seminars: | | | | |
| 1 Interesting | +12 | +7 | +8 | +27 |
| 2 Worthwhile | +12 | +5 | +1 | +18 |
| 3 Demanding | − 3 | −5 | −5 | −13 |
| Did the Working Groups work for you in: | | | | |
| 1 Increasing understanding between doctors and managers | + 9 | +7 | +3 | +19 |
| 2 Improving personal links | + 9 | +6 | +6 | +21 |
| 3 Change your views | 0 | −4 | −8 | −12 |
| Do you feel that this initiative or meetings between doctors and managers should continue: | +12 | +9 | +5 | +26 |
| If so, what form should it take: | | | | |
| 1 Seminar/Lecture | − 1 | +1 | 0 | 0 |
| 2 Group Work | + 6 | +4 | +2 | +12 |
| 3 Combination | +10 | +4 | +3 | +17 |
| 4 Other | − 2 | 0 | 0 | − 2 |
| Do you find the handouts and reports: | | | | |
| 1 Informative | + 8 | +8 | +1 | +11 |
| 2 Useful | + 7 | +3 | +1 | +11 |
| 3 Time Consuming | − 2 | −2 | +3 | 1 |

3. To provide handouts and a post seminar synopsis which enabled people who had been unable to attend to keep up with the rest of the group;
4. To give an overall feedback to participants at the end;
5. To use the criticisms to build for the future.

Interestingly, in the Autumn/Winter of 1990/91 a similar series has been run, again using a seminar followed by group work. Change has continued to occur within Management and within Medicine and this second series occurred during a key time of consultation on an NHS Trust for North Devon where following a sustained campaign by doctors against the Trust, there was then a period of limbo while the Secretary of State made a decision. It was useful to keep momentum and to keep Clinicians and Managers working together through a difficult time of the year. Now, with the Trust agreed to proceed on 1 April 1991, a new Trust Manager, Chairman and Board, this educational initiative has been welcomed by the Northern Devon Trust and seen as a way in which relationships can be furthered in the ever difficult world of the Clinician and the Manager.

